# Treatment of Glioblastoma (GBM) with the Addition of Tumor-Treating Fields (TTF): A Review

**DOI:** 10.3390/cancers11020174

**Published:** 2019-02-02

**Authors:** Denise Fabian, Maria del Pilar Guillermo Prieto Eibl, Iyad Alnahhas, Nikhil Sebastian, Pierre Giglio, Vinay Puduvalli, Javier Gonzalez, Joshua D. Palmer

**Affiliations:** 1 Department of Radiation Oncology, The Ohio State University, Columbus, OH 43210, USA; denise.fabian@osumc.edu (D.F.); Nikhil.Sebastian@osumc.edu (N.S.); 2 Department of Neuro-Oncology, The Ohio State University, Columbus, OH 43210, USA; Pilar.Prieto@osumc.edu (M.d.P.G.P.E.); Iyad.Alnahhas@osumc.edu (I.A.); Pierre.Giglio@osumc.edu (P.G.); Vinay.Puduvalli@osumc.edu (V.P.); Javier.GonzalezAlarcon@osumc.edu (J.G.)

**Keywords:** glioblastoma, GBM, tumor treating fields, TTF

## Abstract

Glioblastoma (GBM) is the most common primary brain tumor. Despite aggressive treatment, GBM almost always recurs. The current standard-of-care for treatment of newly diagnosed GBM has remained relatively unchanged since 2005: maximal safe resection followed by concomitant chemoradiation (CRT) with temozolomide (TMZ), and subsequent adjuvant TMZ. In 2011, the first-generation tumor treating fields (TTF) device, known at the time as the NovoTTF-100A System (renamed Optune), was approved by the Food and Drug Administration (FDA) for treatment of recurrent GBM. The TTF device was subsequently approved as an adjuvant therapy for newly-diagnosed GBM in 2015. The following is a review of the TTF device, including evidence supporting its use and limitations.

## 1. Background

Glioblastoma (GBM) is the most aggressive and common primary brain tumor. Treatment remains challenging, as GBM inevitably recurs despite surgical debulking, radiation and chemotherapy. The current standard-of-care is comprised of maximal safe resection—gross total resection if feasible—followed by chemoradiation (CRT). Radiation therapy consists of 60 Gray (Gy) in 30 fractions over a period of 6 weeks with concomitant daily temozolomide (TMZ), followed by adjuvant TMZ (days 1–5 every 28 days). Median survival ranges from twelve to fifteen months following diagnosis and treatment [1]. The current five-year survival rate is about five percent in the United States [2].

Since 2005, several clinical trials have been conducted attempting to improve outcomes for GBM patients. For example, the Radiation Therapy Oncology Group (RTOG, Philadelphia, PA, USA) 0525 was a phase III trial that compared conventional adjuvant TMZ with dose-dense (dd) TMZ. Despite confirming the prognostic significance of *MGMT* promotor methylation, survival did not improve with dd TMZ [3]. The addition of Bevacizumab in RTOG 0825 demonstrated improvement in progression free survival (PFS), however, it did not yield changes on overall survival (OS) [4,5]. The addition of Everolimus, an oral mammialian target of rapamycin (mTOR) inhibitor, to chemoradiation, increased treatment-related toxicities, and did not have any impact on progression-free survival (PFS) and it even shortened the OS [4]. Additional trials are being conducted to look into the use of checkpoint inhibitors, such as Ipilimumab and Nivolumab (NRG-BN002) and radiation dose-escalation with photon Intensity modulated radiotherapy (IMRT) or Proton Beam Therapy (NRG-BN001) NRG Oncology is a National Clinical Trials Network group created through the efforts of the National Surgical Adjuvant Breast and Bowel Project (NSABP), the RTOG, and the Gynecologic Oncology Group (GOG). Recently, a Phase II Trial of Neoadjuvant TMZ followed by accelerated hypofractionated radiation therapy (60 Gy in 20 fractions) demonstrated a median OS of twenty-two months with a PFS of 13.2 months, comparing favorably to OS previously reported in other clinical trials [6].

In 2011, the United States Food and Drug Administration (FDA) approved a tumor treating fields (TTF) device for treatment of recurrent or refractory GBM. More recently, the FDA approved the TTF device as adjuvant treatment for newly-diagnosed patients after completing standard-of-care surgery and chemoradiation. The National Comprehensive Cancer Network (NCCN) added the TTF device as an option for treatment of newly-diagnosed GBM. Despite FDA approval, skepticism remains regarding this therapy. In this review we discuss the current evidence supporting treatment with the TTF device and its limitations.

## 2. Materials and Methods

We conducted a comprehensive literary investigation utilizing PubMed and Google search engines. Approximately 50 journal articles, newspaper articles, and abstracts were reviewed. Ultimately, 43 sources were selected for relevance and impact. Relevance of topics was selected based on talking points at the 2018 American Society for Radiation Oncology (ASTRO) conference and common questions proposed by the patients and practicing clinicians at our institution.

### 2.1. Tumor Treating Fields Device Proposed Mechanism

The TTF device includes four transducer arrays, each consisting of nine insulated electrodes which are applied to the patient’s scalp to deliver low-intensity, intermediate-frequency (100–300 kHz) alternating electric fields [7,8]. In 2004, a preclinical model demonstrated the inhibitory effect of the TTF device on proliferating cells whereas nonproliferating cells remained unaffected. Treatment with the TTF device is thought to interfere with normal polymerization and depolymerization of microtubules of the mitotic spindle by positioning tubulin dimers further away from the growing end of the microtubules [8]. This leads to mitotic disruption, which leads to mitotic catastrophe and ultimately to mitotic cell death. The investigators demonstrated this by setting up melanoma cell cultures in vitro with TTFs generated by pairs of insulated wires. In cells exposed to TTF, significant inhibition of growth was seen after 24 h exposure. This effect was also seen beyond the exposure time [8]. To explore the effects of TTF on molecular processes, the investigators used time-lapse microphotography. In cells treated with TTF, mitosis began normally, but was prolonged [8]. Additionally, a quarter of cells in the TTF cultures were destroyed during the formation of the mitotic cleavage furrow [8]. Finally, nuclear rotation was seen in the TTF cultures [8]. The investigators explain that microtubules in dividing cells have electric dipole moments, which may be altered by the forces exerted by TTF [8]. They showed this by comparing the movement of cellular microtubules by fixing the cells after 24 h of TTF vs. no treatment; when the fixed cells were viewed under fluorescence microscopy, more than 50% of the TTF treated cells had abnormal mitosis compared to less than 5% of the control cells [8]. The investigators defined two mechanisms of action: (1) disruption of the polar tubulin molecule orientation, pushing tubulin dimers further than 14 nm away from the growing end of the microtubule and thereby interfering with proper microtubule assembly; and (2) cell destruction by pulling of all intracellular and polar particles towards the mitotic cleavage furrow, resulting in a pile-up that interferes with cytokinesis [8].

Further laboratory studies demonstrated similar cancer growth inhibition in multiple in vitro cell lines and animal tumor models. A single-arm pilot clinical trial was performed in ten patients shortly thereafter [9]. Novocure Ltd. (St. Helier, NJ, USA) manufactured a clinical TTF device, named Optune (formally Novo TTF-100A, St. Helierl, NJ, USA). It utilizes electrodes configured as a cap that is placed on the patient’s shaved scalp and powered by a battery package [10]. At present, the proposed mechanism also includes interference with Septin fibers of proliferating cells as well as endoplasmic reticulum stress leading to cellular stress and autophagy [11,12]. This demonstrates that the effect of TTF on interactions of intercellular may be more complex than originally thought; similarly, the effects on many other intracellular proteins are unknown. Further studies are warranted in both cancer and normal cell lines.

### 2.2. Treatment of Glioblastoma with Tumor Treating Fields

The first phase III clinical trial testing the TTF device (EF-11) was published in 2012 and included 237 patients with recurrent GBM [13]. This study compared the first generation of the TFF device alone—worn 18–24 h a day—to chemotherapy of the treating physician’s choice. The median survival was 6.6 vs. 6.0 months (*p* = 0.27) for patients subject to treatment utilizing the TTF device or chemotherapy, respectively. Although the trial did not demonstrate its primary endpoint of improved overall survival, efficacy was similar to commonly used chemotherapy regimens. Given the localized nature of the treatment, a lower toxicity profile and better quality of life—including improved cognition and emotional well-being—were reported in the TTF arm. These patients also experienced less chemotherapy-related side effects, such as hematologic toxicity, gastrointestinal adverse events, and infections. Further analysis found that compliance with therapy was linked to outcomes; more specifically device compliance above 75% correlated with improved OS [14]. It is important to note that the trials were not powered to look into these subgroup analyses. The limitations of the study included absence of a placebo control arm and a heterogeneous patient population who had received various chemotherapy treatments prior to the trial. The results from this trial lead to the 2011 FDA approval of the first generation of the TFF device for treatment of patients with recurrent GBM or GBM that has not responded to traditional therapy [15]. Approval of this device was contingent on Novocure conducting additional studies and clinical trials to demonstrate device efficacy [16].

Following the trial, the Patient Registry Data set (PRiDe)—a registry of all patients with recurrent GBM who received therapy with the TTF device—was used to analyze the clinical outcomes of TTF device use in 91 cancer centers across the United States [17]. Compared to patients in the EF-11 trial, the 457 recurrent GBM patients in the PRiDe data set were more likely to use the TTF device for their first recurrence (33% vs. 9%). Median OS was improved in clinical practice when compared to the EF-11 trial (9.6 months vs. 6.6 months, hazard ratio (HR) 0.66, *p* = 0.003). As seen previously, device compliance rate of 75% or greater was associated with a significantly improved overall survival when compared to device compliance rate of less than 75% (7.7 vs. 4.5 months, *p* = 0.042). There were no significant adverse events, and the most common side effect was skin reaction [17].

In 2009, a multi-center phase III clinical trial lead by Dr. Roger Stupp was initiated for newly diagnosed GBM, studying the addition of TTF device treatment to maintenance TMZ (EF-14 trial) [7]. This study randomized 695 GBM patients in a 2:1 format to receive TTF device treatment plus maintenance TMZ or TMZ alone, following standard-of-care surgery and concurrent chemoradiotherapy. Patients with a Karnofsky performance score (KPS) of less than seventy, evidence of progressive disease following chemoradiotherapy, infratentorial tumor location or severe comorbidities were excluded from the study. An interim analysis presented in 2015 demonstrated improved PFS (7.1 months in the TTF device plus TMZ group and 4.0 in the TMZ alone group (*p* = 0.001) [7]. The final study was published in 2017, which reported an improved median overall survival of 20.9 months in the TTF device plus TMZ group vs. 16.0 months in the TMZ only group (*p* < 0.001). Of note, the reported survival times were measured from time of randomization, which was done after completion of chemoradiation, and which was about 3.8 months from original diagnosis. About half of patients who received treatment with the TFF device experienced mild to moderate skin toxicity [7]. Accordingly, the FDA approved the use of the TTF device for use in newly diagnosed GBM on 5 October 2015 [15]. Both Phase III clinical trials using TTF for GBM are summarized in Table 1.

This publication lead to the National Comprehensive Cancer Network (NCCN) adaptation of TTF treatment to the Clinical Practice Guidelines in Oncology for Central Nervous System. Additional analysis of the EF-14 trial has shown that the overall survival (OS) is improved at five years, regardless of other prognostic factors, such as age, performance status, extent of resection, and neurologic status. An abstract presented at ASTRO 2018 showed that that use of the TTF device and TMZ improved overall survival out to five years in all three recursive partitioning analysis (RPA) classes [18].

As expected, further clinical investigations using TFF are well underway (Table 2). In November 2018, a phase II trial opened comparing a combination treatment with the TTF and nivolumab with or without ipilimumab in patients with bevacizumab-naive recurrent GBM [19]. The trial is expected to close in 2021 and has an accrual goal of sixty (60) patients. Table 2 is a summary of ongoing trials using TTF in GBM. Research on utilization of TTF in cancer care is also not limited to GBM. A phase 2 pilot study on the safety and efficacy of the TTF device concomitant with pemetrexed and cisplatin or carboplatin in malignant pleural mesothelioma (STELLER) recently closed in April 2018. In this study, 150 kHz electrode arrays were placed on the thorax of patients with previously treated malignant pleural mesothelioma in addition to chemotherapy. The preliminary efficacy will be compared to historical cohorts [20]. TFF devices are being explored in disease sites such brain metastasis secondary to non-small cell lung carcinoma, ovarian carcinoma, pancreatic carcinoma, meningioma and even as an alternative to prophylactic cranial irradiation in small cell lung cancer [21,22].

### 2.3. Existing Skepticism

The TFF device has been described as “polarizing” amongst neuro-oncology experts. To date, the TTF device is not covered by Medicare or many other insurance companies, on the grounds that the therapy is still experimental. More recently, Novocure has applied for a reconsideration request, which has been accepted for local coverage determination [15].

Despite FDA-approval in the United States (US), existing skepticism regarding the use of the TTF device persists. Primary criticism includes the unblinded nature of the TTF clinical trials. A “sham” device—to better discern a potential placebo-effect of wearing the device—wasn’t used. The lack of blinding becomes more of an issue when the primary outcome is PFS. Other concerns arise from the lack of understanding of the TTF device’s manner of operation, specifically across a variety of tissues and in combination with other treatments [23]. Furthermore, the fact that randomization in the EF-14 trial occurred over two months after diagnosis suggests a selection bias of patients who did not have progression after the initial treatment and would therefore likely have better survival regardless. Hence, it is difficult to consider the device as “standard-of-care” for future patients who may either: (1) be receiving a shorter course of radiotherapy; or (2) experience an interruption chemotherapy during initial treatment [23].

Several of these points were addressed during a round table discussion of the EF-14 trial at ASCO 2015. This meeting included 5 neuro-oncology experts who did not participate in the trial [21]. In response to the time of randomization question, the EF-14 trial compared favorably in efficacy to other GBM trials when adjustments were made for this difference. Specifically, the improvement in the 2-year survival rate, which was 43% with TTF vs. 29% without TTF, along with median overall survival, (19.6 months with TTF device treatment vs. 16.6 months without TTF device treatment, HR = 0.744, *p* = 0.0038) were considered clinically meaningful [21]. Omission of a placebo-control device in the trial was also a topic of consideration. Several arguments were made in support of this decision, including that: (1) the panel thought it was unlikely that an objective endpoint like OS would be a result of placebo effect; (2) the magnitude of benefit was beyond what would be expected for a placebo effect (HR of 0.75 for OS); and (3) previous trials that lacked a placebo, such as RTOG 0525, did not show improved survival [21]. Ultimately, the group decided that that the TTF device should be considered a treatment option for patients with GBM who were willing to undergo therapy and did not have contraindications. Furthermore, future studies were recommended to identify which subset of patients benefits most from TTF therapy [21].

Despite criticism, most medical device evaluations traditionally lack randomized control groups [24]. Although this may be attributed to the lesser sophistication of clinical trial design by device manufacturers, it’s also likely secondary to properties of the device. In this case, patients sense heat from the TTF device, which would be impractical for a sham device. Furthermore, ethical issues arise. For example, it would be difficult and unethical to observe a placebo heart valve replacement [24]. Similarly, it is argued that having a patient shave their head and wear a sham cap for greater than eighteen hours a day would also be unethical. Nevertheless, long-term observational studies will be necessary to observe device efficacy.

### 2.4. Safety

Use of a TFF device was not associated with systemic toxicity in either the EF-11 or EF-14 trials. In the EF-11 trial, typical systemic side effects were not seen; whereas in the EF-14 trial, systemic side effect rates did not show a significant difference from the TMZ-alone cohort (48% vs. 44%) [7,13]. Some events in EF-14 had a slightly higher incidence in the TTF group, which was attributed to longer use of TMZ in this group due to delayed progression [7]. A common toxicity was a moderate skin reaction on the scalp below the transducers; dermatitis was observed in 16% of patients wearing the TFF device on EF-11 and 52% of patients in EF-14 [7,13]. Severe skin toxicity was seen in 2% of patients on EF-14 [7]. TFF therapy did not increase the incidence of seizures in either study. The mild to moderate skin reaction patients may experience is reversible and does not require treatment discontinuation [25]. Recently, a phase I clinical trial of 10 patients exploring TTF device treatment with concomitant radiation therapy reported preliminary results of similar rate of TTF-related skin toxicity (40%) to that of the EF-14 trial [26].

### 2.5. Compliance and Quality of Life

The efficacy of the TFF device has been correlated with patient compliance, and a compliance rate of over 75% has been linked to higher OS [14]. A recently published subgroup analysis showed that a threshold value of 50% compliance, defined as percent usage per month, improved PFS (HR = 0.70) and OS (HR 0.67) [27]. This was seen independent of gender, extent of resection, *MGMT* status, age or performance status [27]. Interestingly, patients with a compliance rate of >90% showed a prolonged median survival of 24.9 months and a five-year survival rate of 29.3% [27].

Consequently, patients need to wear the TFF device continuously with minimal interruption. This inevitably requires lifestyle modifications. Enrollment in the trial was self-selecting and the cohort was not representative of the entire GBM population. Some patients may find the device confining, as it potentially interferes with daily activities. Despite improved survival in the EF-14 trial, patients in clinic may be reluctant to use the TFF device for social or cultural reasons [22]. Others may be averse to shaving their heads. Studies on chemotherapy-induced alopecia have shown that the psychosocial impact of hair loss can be devastating [28]. Quality of life is certainly a priority for this patient population.

A planned interim analysis of the EF-14 trial analyzing health-related quality of life (HRQoL) was performed on 315 patients. HRQoL was slightly improved for patients in the TTF group at six months; physical and social functioning showed no difference at nine and twelve month time points. Patients in the TTF group complained of itchy skin. There was no preliminary evidence that HRQoL, functional status, or cognitive functioning was altered by use of TTF [29]. A final analysis, published in 2018, found that HRQoL did not differ significantly between the two arms of the trial with the exception of itchy skin in the TTF arm [29]. The analysis found that deterioration-free survival was longer in patients receiving TTF therapy for global health, physical and emotional functioning [29]. However, it is important to note that adherence to the HRQoL survey questionnaire decreased significantly over time; survey completion-rate was 91.9% at baseline, 65.8% at three months, and 41.7% at twelve months [29]. Remaining HRQoL scales not previously reported were subjected to an exploratory analysis, which was presented at ASTRO 2018. This study reported a larger proportion of patients having stable/improved bladder control and diarrhea when compared to baseline. The deterioration-free survival for diarrhea, future uncertainty, and headaches was delayed in patients receiving TTF and TMZ treatment when compared to treatment consisting of TMZ alone (HR 0.68, *p* < 0.001). No negative impact on HRQoL was seen in this study [30].

Efforts are underway to improve convenience of utilizing the TTF device. The first-generation Optune TTF device weighed 6 pounds and consisted of a field generator, transducers, lithium batteries and a carry bag. In 2016, a second-generation TFF device weighing only 2.7 pounds was approved [31]. The goal of the second-generation TFF device was to improve patient convenience and compliance. Additionally, the newer device contains an objective log that records treatments compliance [31]. There is hope that the growing popularity of wearable devices, such activity trackers and other smart devices, may encourage patients to be more open about wearing the TTF device [22].

### 2.6. Cost of the Device

As mentioned previously, the specific payments for this device have not yet been established by Medicare and other insurance providers. Medicare’s Ambulatory Payment Classification covers the technical component of the device; Medicare’s Physician Fee reimburses the professional component of therapy [29]. The total monthly therapy cost is about $21,000, which translates to about $86,000 for the average patient using the device for 4.1 months [30]. Novocure’s executive chairman William F. Doyle reported that the company had been providing the device for free for to patients without health insurance in a 2015 New York Times Article [32].

There have been few studies evaluating the cost-effectiveness of TTF. A French group measured the cost-effectiveness of the device and measured it to be “far beyond conventional thresholds due to the prohibitive announced cost of the device.” [33]. This report suggested that there is 0% chance of achieving the cost-effectiveness threshold, which was chosen arbitrarily, of €100,000 ($114,213 in US dollars) per year life gained [33]. Total cost for TTF therapy and conventional therapy strategies were €243,141 and €57,665, respectively. After applying a 4% annual discount according to French national guidelines, the analysis resulted in a total incremental-cost effectiveness ratio of €596 411 per year life gained [33]. A US group presented an abstract at the America Association for Cancer Research (AACR) meeting in 2018 projected that TFF plus TMZ compared to TMZ alone resulted in an undiscounted mean survival of 1.8 life years based on 5 survival results of EF-14 trial [34]. The incremental cost effectiveness ratio based on this survival was $150,638 per life year gained and $198,032 per quality life year gained. This abstract reported a high probability of cost-effectiveness at a threshold of $200,000 [34]. Regardless, the device is certainly expensive and the cost-effectiveness threshold will likely vary considerably between, and even within, countries [35].

How does TTF therapy compare to other novel cancer therapies available in the US? The CheckMate 067 trial evaluating dual checkpoint inhibitors nivolumab and ipilimumab in patients with advanced melanoma showed an increase in PFS from 6.9 months with nivolumab alone to 11.5 months with combination therapy [36]. In 2015, the estimated cost per patient per year for nivolumab was $103,220 and ipilimumab was $158,252. The combination therapy was estimated to run $295,566 [37]. The cost-effectiveness of nivolumab in patients with advanced renal cell carcinoma treated in the US found this treatment to have a 91.7% probability of being cost-effective at a $150,000 threshold per quality adjusted life year. Other treatments like chimeric antigen receptor (CAR) T-cell therapy are costly with a price tag of $475,000 for Tisagenlecleucel and $373,000 for Axicabtagene ciloleucel [38].

Currently, the average direct cost of therapy for GBM patients in the US is about $8500 per month, and there is regional variability [39]. Mayo clinic reported a median total direct cost of $91,000 from 1987 to 1992, before the routine use of TMZ: radiotherapy and imaging cost contributed the most to this number [39,40]. It has since been reported that the addition of TMZ, in both the concomitant and adjuvant settings, has led to an eightfold increase to the direct cost of GBM therapy [39,41]. Nevertheless, administration of TTF therapy significantly increases the overall cost of GBM treatment.

## 3. Discussion

Since the publication of the Stupp trial in 2005, there have been minimal advancements in GBM therapy. Trials that studied targeted molecular inhibitors, immunotherapy agents, vascular growth factor inhibitors, and radiotherapy dose escalation have fallen short. The EF-11 and EF-14 trials have demonstrated that TTF therapy is a promising alternative to chemotherapy in patients with recurrent GBM and adjuvant therapy for patients with newly diagnosed GBM. Most interestingly, the EF-14 trial reported an improved median overall survival of 20.9 months in the TTF plus TMZ group vs. 16.0 months in the TMZ only group. This led to the NCCN endorsement of routine adaptation of TTF as an upfront therapy for patients with newly diagnosed GBM. Since clinical implementation, there have been several reviews on TTF therapy for GBM [21,42]. Given the amount of newer studies done within the last year on this topic, especially in the realm of quality-of-life and cost-effectiveness, we conducted an updated review.

The implementation of the device has polarized many neuro-oncology experts. Some are enthusiastic about this development while others remain skeptical, perhaps understandably. There is a general lack of understanding of the TTF device’s mechanism of action beyond what is proposed at the cellular level. Of note, most of the pre-clinical data used to drive the trials was pioneered by Novocure scientists, like Dr. Eilon Kirson. Additionally, the absence of a placebo-control “sham” device in the trials makes some experts less eager to recommend the therapy to their patients. However, it has been discussed that the inclusion of such a device in the trial would have created an ethical dilemma. Moreover, for better or worse, a more lenient precedence for medical devices exists: these devices become approved without randomized, placebo-controlled studies. Again, it is noted that both the EF-11 and EF-14 trials were sponsored by Novocure. Despite these considerations, the EF-14 trial certainly produced compelling results and clinicians echo the phrase “it is difficult to argue with overall survival.”

One of the attractive properties of TTF therapy is the minimally-invasive nature and lack of systemic side-effects. This is especially important in the realm of recurrent disease, where patients undergo a variety of treatments from chemotherapy to additional surgery and/or re-irradiation. There are lifestyle drawbacks, as the device needs to be worn as continuously as possible, especially given the recent analyses showing correlation between device compliance and OS. Device compliance of >90% notably correlated with a 5-year OS reaching 30%. It is reassuring that most of the quality of life studies have reported similar results between patients who received TTF and those who did not. Neither health-related quality of life, functional status, nor cognitive functioning have been shown to be altered by TTF use.

The cost of therapy continues to be high, which may be prohibitive for certain patients. Willingness-to-pay for the device varies significantly between countries and even between regions. However, it is important to consider that other novel cancer therapies—such as dual checkpoint blockade with nivolumab and ipilimumab and CAR T-cell therapy—also carry a very high price tag. Hopefully prices will become more manageable as these therapies mature and become more widespread. It’s important to make prices manageable for patients while also maintaining incentive for product innovation.

An additional obstacle is that the device is not routinely available in many centers. An electronic survey sent to an international group of radiation oncologists, neurosurgeons, and neuro-oncologists between January 2015 and July 2015 found that only 41% of surveyed practitioners had TTF available to offer their patients [43].

## 4. Conclusions

Ultimately, long-term, real-world results are necessary to make clinicians more comfortable with the device. Furthermore, positive clinical trial results in disease sites other than GBM may make the device more acceptable and familiar. It remains important to define which subset of patients benefit the most from TTF therapy, through identifying markers that predict improved outcome with TTF therapy. Reluctant patients may be more willing to try the device if it is more convenient and less intrusive to daily life. Further industry efforts to improve the device will help with patient compliance, such as the lower weight in second generation devices. Finally, it is important to define the benefits and potential toxicities of TFF therapy in combination with radiation therapy and chemoradiation therapy via larger, randomized clinical trials. Should we expect to hear much more about TTF therapy in oncology? We’ll bet our best hat.

## Figures and Tables

**Table 1:** Completed clinical trials using tumor treating fields (TTF) for glioblastoma (GBM), published ^1^.

Trial Name	Patient Cohort	Number of Patients	Study Design	Intervention	Endpoints	Toxicity	Ref.
EF-11 (NCT00379470)	GBM, progressed on prior therapy	237	Prospective, randomized (1:1) Phase III	Standard: best available CHT alone; Experimental: TTF alone (20–24 h/d)	Median OS 6.6 vs. 6.0 months (primary endpoint); 1-y OS 20% vs. 20%; 6-month PFS 21.4% vs. 15.1% (NS)	Severe adverse events 6% vs. 16% (*p* = 0.022). TTF-related adverse events were mild (14%) to moderate (2%) skin rash.	[12]
EF-14 (NCT00916409)	Newly diagnosed GBM after completion of concurrent TMZ and RT.	695	Prospective, randomized (2:1) Phase III	Standard: Maintenance TMZ (150–200 mg/m^2^/d for 5 days every 28 days for 6–12 cycles); Experimental: Maintenance TMZ with TTF (>18 h/d)	Median PFS 20.9 months vs. 16.0 months (*p* < 0.001)	Grade 1–2 skin toxicity 52% vs. 0%.	[6]

^1^ Abbreviations: GBM: glioblastoma multiforme, CHT: chemotherapy, TMZ: temozolomide, TTF: tumor treating fields, OS: overall survival, PFS: progression-free survival.

**Table 2:** Ongoing trials using TTF for GBM as of December 2018 ^1^.

ClinicalTrials.gov Identifier	Patient Cohort	Study Design	Intervention	Primary Outcome Measures	Secondary Outcome Measures	Estimated Enrollment	Duration	Institution
NCT03430791	Bevacizumab-naïve, recurrent GBM	Prospective, randomized Phase II	TTF + nivolumab +/− ipilimumab	ORR	-	60	Nov 2018–Aug 2021	Miami Cancer Institute
NCT03405792	Newly diagnosed GBM after resection and CRT.	Phase II, single arm	TMZ + TTF + pembrolizumab	PFS	AEs, OS, Augmentation of TTF immune reaction	29	Feb 2018–Feb 2023	University of Florida
NCT03477110	Newly diagnosed GBM	Phase I, single arm, single institution	CRT + TTF (up front at initiation of CRT)	TTF-discontinuation rate due to toxicity	PFS, PS, EFS	35	May 2018–Mar 2020	Thomas Jefferson University
NCT02663271	Bevacizumab-refractory recurrent GBM	Phase II multicenter, single-arm	TTF + bevacizumab	PFS	AEs, KPS/MMSE change from baseline, imaging response	18	Aug 2016–Mar 2019	University of Florida, Washington University
NCT02743078	Bevacizumab-refractory recurrent GBM	Phase II, multicenter, single-arm	TTF + bevacizumab	OS	PFS, ORR, AEs	85	Apr 2017–Aug 2022	Multiple
NCT01894061	Bevacizumab-naïve, recurrent GBM	Phase II, single-arm	TTF + bevacizumab	PFS	ORR, AEs, neuro-cognition, QOL	40	June 2013–May 2019	Case Western, Cleveland Clinic, University of Cincinnati
NCT03223103	Newly diagnosed GBM, after CRT	Phase I single-arm, single-institution	TTF + mutation-derived tumor vaccine	DLT	Toxicity, PFS, OS, ORR	20	Mar 2018–May 2020	Mt. Sinai
NCT02903069	Newly diagnosed GBM	Phase I, multicenter	TTF + proteasome inhibitor	MTD/DLT	AEs, OS, PFS	72	Apr 2016–May 2020	Multiple

^1^ Abbreviations: GBM: glioblastoma multiforme, TTF: tumor treating fields, ORR: objective response rate, OS: overall survival, PFS: progression-free survival, TTP: time to disease progression, QOL: quality of life, AEs: adverse events, CRT: chemoradiation, EFS: event-free survival, MTD: maximum tolerated dose, DLT: dose-limiting toxicities.
